# OsGatB, the Subunit of tRNA-Dependent Amidotransferase, Is Required for Primary Root Development in Rice

**DOI:** 10.3389/fpls.2016.00599

**Published:** 2016-05-02

**Authors:** Cheng Qin, Linming Cheng, Huanhuan Zhang, Meiling He, Jingqin Shen, Yunhong Zhang, Ping Wu

**Affiliations:** ^1^Research Centre for Plant RNA Signaling, College of Life and Environmental Sciences, Hangzhou Normal UniversityHangzhou, China; ^2^The State Key Laboratory of Plant Physiology and Biochemistry, College of Life Science, Zhejiang UniversityHangzhou, China

**Keywords:** OsGatB, tRNA-dependent amidotransferase, tRNA^Gln^, Primary root, Rice

## Abstract

A short-root rice mutant was isolated from an ethyl methane sulfonate-mutagenized library. From map-based cloning strategy, a point mutation, resulting in an amino acid change from proline to leucine, was identified in the fourth exon of a glutamyl-tRNA (Gln) amidotransferase B subunit family protein (*OsGatB*, LOC_Os11g34210). This gene is an ortholog of *Arabidopsis GatB* and yeast *PET112*. GatB is a subunit of tRNA-dependent amidotransferase (AdT), an essential enzyme involved in Gln-tRNA^Gln^ synthesis in mitochondria. Although previous studies have described that cessation in mitochondrial translation is lethal at very early developmental stages in plants, this point mutation resulted in a non-lethal phenotype of smaller root meristem and shorter root cell length. In the root, *OsGatB* was predominantly expressed in the root tip and played an important role in cell division and elongation there. OsGatB was localized in the mitochondria, and mitochondrial structure and function were all affected in *Osgatb* root tip cells.

## Introduction

Mitochondria are the powerhouses of eukaryotic cells, ATP is generated there, and then used to support cell growth and maintenance (Goh et al., 2004). Plant mitochondria contain about 322 proteins (Huang et al., 2013), and most are encoded by the nuclear genome and translocated to the mitochondria after being synthesized and modified in the cytoplasm (Rehling et al., 2003). However, mitochondria also have their own genome and can synthesize proteins, which also play important roles in maintaining mitochondria structure and function (Unseld et al., 1997).

There are 20 aminoacylated transfer-RNAs (aa-tRNAs) involved in protein synthesis. Most aa-tRNAs are formed in a direct pathway by aa-tRNA synthetases, which acylate each tRNA species with its cognate amino acid. However, four aa-tRNAs can also be formed through a different indirect pathway, one of which is Gln-tRNA^Gln^ (Sheppard et al., 2008). In the indirect pathway, a tRNA-dependent amidotransferase (AdT) is needed to convert the misacylated Glu-tRNA^Gln^ to Gln-tRNA^Gln^.

As one subunit of AdT, GatB is located in mitochondria and involved in protein synthesis there. In *Saccharomyces cerevisiae*,it was reported that Gln-RNA^Gln^ in mitochondria is synthesized by an AdT, one subunit of which is the gatB ortholog, PET112 (Frechin et al., 2009). It is also known that PET112 is encoded in the nuclear genome and is essential for mitochondrial function (Mulero et al., 1994). In *Arabidopsis*, the formation of Gln-tRNA^Gln^ in mitochondria is also through the indirect pathway. The GatB ortholog, AtGatB is transported into mitochondria and form the functional AdT (Pujol et al., 2008). It was recently reported that another subunit of AdT, *GatA* knockdown in mouse and human cultured cells can reduce the levels of the enzyme AdT, and strongly impaired mitochondrial translation *in vivo* (Echevarría et al., 2014). These results strongly indicated that mitochondrial AdT could well exist and was essential for synthesis of Gln-tRNA^Gln^, and therefore protein synthesis, in yeast and mammalian mitochondria. Despite these advances, however, our understanding of the function of GatB in plant development, especially root development, is still at an early stage.

Here, we isolated and analyzed a short-root rice mutant obtained from EMS mutagenesis. Through a map-based cloning approach, the mutated gene was identified as *OsGatB*, encoding a subunit of mitochondrial AdT, which is involved in formation of Gln-tRNA^Gln^ in mitochondria. We showed that, in the root, OsGatB was predominantly expressed in the root meristem where it was required for cell division. We also showed that OsGatB was localized in the mitochondria. Importantly, mitochondrial structure and function were both affected in *Osgatb* root tip cells.

## Materials and methods

### Plant materials and growth conditions

The rice (*Oryza sativa*) mutant *Osgatb* was isolated from an ethyl methane sulfonate (EMS)-mutagenized population of a *Japonica* cultivar “Shishoubaimao.” Culture solution was as described by Yoshida et al. (1976). Seedlings were grown at 30/22°C (day/night) in a growth chamber, with relative humidity of 60–70% and photoperiod of 12 h, with a photosynthetic photon flux density of ~200μmol photons m^−2^s^−1^.

### Microscopic analysis

A stereomicroscope (MZ95, Leica, Bensheim, Germany) with a color charge-coupled device (CCD) camera was used to examine and photograph the plant roots. For microscopic analysis, root tips from 10-day-old plants were fixed overnight at 4°C in 2.5% glutaraldehyde (0.1 M sodium phosphate buffer, pH 7.2), and then washed three times for 30 min in the same buffer. Root samples were then fixed in OsO_4_ for 4 h at room temperature and washed for 30 min in the same buffer. Samples were dehydrated through an ethanol series (30, 50, 70, 80, 90, 95, 100, 100, and 100% ethanol), 30 min in each and then infiltrated through ethanol. The samples were embedded in pure Spurr's resin, and polymerized overnight at 70°C. Semi-thin sections (2 μm thick) were made using glass knives on a power Tome XL microtome (RMC-Boeckeler Instruments, Tucson, AZ, USA) and stained with 0.1% methylene blue for 3 min at 70°C. The samples were rinsed with distilled water and visualized with a Zeiss Axiovert 200 microscope with a color CCD camera (Zeiss, Jena, Germany). The average cell length of the maturation zone of primary root was obtained from at least 16 root longitudinal sections. The average root length of WT or *Osgatb* mutant stands for the average of measured values of at least 10 different seedlings.

### Map-based cloning of *OsGatB*

The *Osgatb* mutant (*japonica*) was crossed with Kasalath (*indica*) to map the *OsGatB* gene. The F_1_ seedlings showed WT phenotype and F_2_ seedlings showed a segregation ratio of 3:1 (132:32, χ^2^ = 1.97 < $\chi_{0.05}^{2}=$ 3.84; *P* > 0.05), suggesting that the mutant phenotype was related to a single recessive nuclear gene. For map-based cloning of the *OsGatB* gene, 2368 F_2_ mutants were selected from the F_2_ population. After fine mapping, *OsGatB* was localized in the region of 73 kb on chromosome 11, between markers STS11g19271K and STS11g19344K. The primers for STS11g19271K were 5′-AGCTGGATTTTGACGCAGAGA-3′ and 5′-ATGGGATTTCGAGGACTATGATG-3′, and those for STS11g19344K were 5′-AAAAGCAACTAAAAACACCATA-3′ and 5′-TATTTCGATTTTGCTATATCTCAC-3′. Based on the mutant phenotype, *OsGatB* was selected as a candidate gene among the several putative proteins encoded by the 73 kb DNA region. Genomic DNA of the gene was amplified by PCR from *Osgatb* mutant and WT plants for sequence analysis.

### Complementation test

The 7.3 kb genomic DNA, including 3360 bp promoter of *OsGatB* before the ATG and the coding region of *OsGatB* gene, was cloned into the binary vector pCAMBIA1300 using BamHI and XbaI. The construct was transformed into *Osgatb* as described by Chen et al. (2011). *OsGatB* primers were 5′-AAGGATCCAAGCTCC TTGTTTGCCTTATACTC-3′ and 5′-ATCTAGAAACTATTG GCCTTCAATTTCTCCCC-3′.

### Construction of pOsGatB:OsGatB–GUS

To develop the pOsGatB:OsGatB–GUS vector, the same 7.3 kb genomic DNA described above, including 3360 bp promoter of *OsGatB* before the ATG and the coding region of *OsGatB* gene was cloned into the binary vector pCAMBIA1300NH-plus GUS using HindIII and BamHI. *OsGatB* -specific primers were 5′-AAAAAGCTTAAGCTCCTTGTTTGCCTTATACTC-3′ and 5′-AAAGGATCCAACTATTGGCCTTCAATTTCTCCCC-3′. The construct was also transformed into *Osgatb* as described by Chen et al. (2011).

### GUS staining

Histochemical GUS analysis was carried out following the instructions of Jefferson et al. (1987). Plant samples were first fixed in ice-cold 90% acetone for 20 min. After three washes in rinse solution [50 mM Na_2_HPO_4_, 50 mM NaH_2_PO_4_, 0.5 mM K_3_Fe(CN)_6_, and 0.5 mM K_4_Fe(CN)_6_], the tissues were infiltrated with staining solution (rinse solution with 1 mM X-Gluc) under vacuum and subsequently incubated at 37°C for 6 h. The stained tissues were cleared of chlorophyll in an ethanol series and observed under a light microscope.

### Subcellular localization analysis

To confirm that the location of the OsGatB protein was in mitochondria, the coding region of *OsGatB* was cloned into the binary vector pCAMBIA1300-35S–sGFP using SacI and BamHI. PDS-1000/He gene gun (Bio-Rad, Hercules, CA, USA) was used to transform the constructor into onion epidermal cells as described by Carrie et al. (2009). The fluorescent dye MitoTracker Orange (CMTMRos, Invitrogen, Carlsbad, CA, USA) was used as a positive control for mitochondrial localization. Fluorescence was visualized by Zeiss LSM 510 confocal microscope. *OsGatB*-specific primers were 5′-AAAGAGCTCATG GCACTGACCCTT-3′ and 5′-AAAGGATCCACTATT GGCCTTCAATTTCTCC-3′.

### Quantitative real-time PCR

Ten-day-old seedlings were used for total RNA extraction. Then, the first-strand cDNA was synthesized using a SuperScript VILO cDNA Synthesis Kit (Invitrogen). For quantitative real-time PCR, a LightCycler480 machine (Roche Diagnostics, Mannheim, Postfach, Germany), and LightCycler 480 SYBR Green I Master were used. The amplification program was 95°C for 10 s, 58°C for 10 s, and 72°C for 20 s. Triplicate quantitative assays were performed. Relative expression level of *OsGatB* mRNA was calculated using the formula 2^−Δ*ΔCt*^ and normalized to actin mRNA. The gene-specific primers for *OsGatB* were 5′-GGTCACAAAGGC GTTCTGCTCGTG-3′ and 5′-AATGGGAATGTC AAACTGCGAAAT-3′.

### TEM

For TEM analysis, the primary roots of seedlings were incubated with 2.5% glutaraldehyde and then with 1% OsO_4_. After that, samples were dehydrated and finally embedded in pure Spurr's resin. Ultrathin sections of the samples were processed, and placed finally onto copper grids. After staining with uranyl acetate and Reynolds' lead citrate, the samples were photographed with a JEM-1010 (JEOL, Tokyo, Japan).

### Measurements of ATP level

For measurement of ATP level in plants, 250-mg samples were ground and incubated with trichloroacetic acid. Then the extracted ATP was measured using a bioluminescent assay kit (Sigma-Aldrich, St Louis, MO, USA).

### Statistical analysis

The significance of differences between data was calculated using Student's *t*-test. Significant differences were indicated with an asterisks (^**^), *P* < 0.01.

## Results

### Isolation of the *OsGatB* mutant

**Figure 1:**
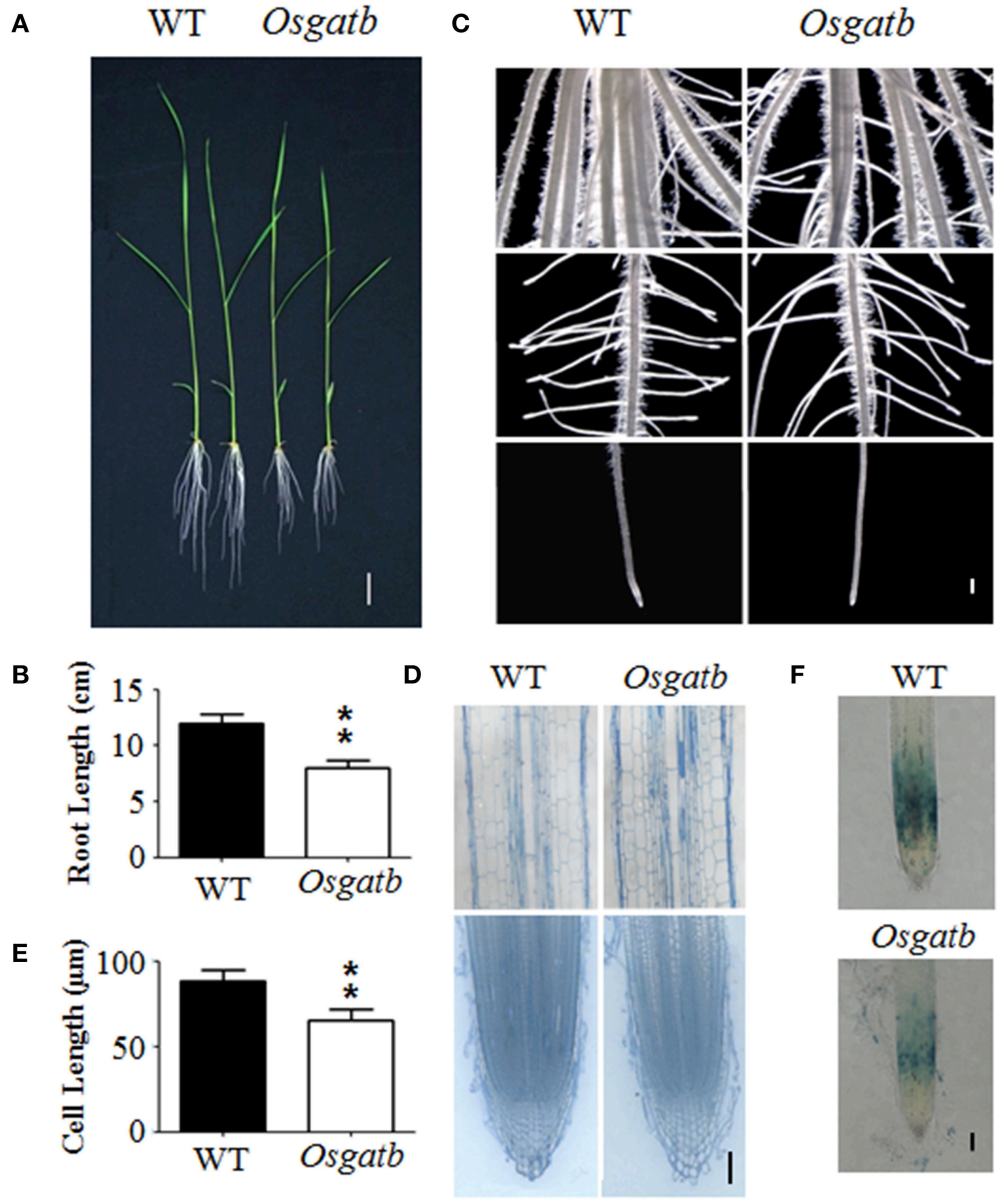
**Phenotype of the *Osgatb* mutant. (A)** Phenotype of 10-day-old seedlings of the WT and *Osgatb* mutant. Bar = 2 cm. **(B)** Primary root length of WT and *Osgatb* mutant. Asterisks show significant differences between WT and mutant. ^**^P < 0.01. **(C)** Stereomicroscope analysis of WT and *Osgatb* mutant. Bar = 1 mm. **(D)** Longitudinal sections of mature region (upper) and root tip (lower) of WT and *Osgatb* mutant. Bar = 200μm. **(E)** Root cell length of WT and *Osgatb* mutant. Asterisks show significant differences between WT and mutant: ^**^P < 0.01. **(F)** GUS staining result of OsCYCB1;1–GUS in WT and *Osgatb* seedlings, the expression region of OsCYCB1;1 in *Osgatb* is smaller than that in WT. Bar = 200 μm.

In order to study the mechanism of rice primary root development, we screened an EMS-mutated rice library (*O. sativa* ssp*. japonica* cv. Shishoubaimao), which contained around 1000 individual M_2_ lines. The seeds were germinated and grown in a nutrient solution (Yoshida et al., 1976). Finally, we isolated a mutant with a short root phenotype (Figures 1A,B). The mutant was named *Osgatb*, and is described later. The mutant showed retarded primary root growth, but growth of adventitious roots, lateral roots and root hairs were the same as for the wild type (WT; Figure 1C), so did shoot length (Figure 1A, Figure S1). This indicated that OsGatB affected only primary root growth.

To further determine the reason for the defect in primary root growth of the mutant, cell elongation and division were examined. Root longitudinal sections were performed to determine the primary root cell length, and showed that cells of *Osgatb* were shorter than of the WT (Figures 1D,E). However, root structure in the root apical meristem seemed normal in *Osgatb* (Figure 1D). In order to check the primary root cell division, *OsCYCB1,1:GUS* was crossed into *Osgatb*, and the expressing region of Os*CYCB1,1:GUS* in *Osgatb* was smaller than in the WT (Figure 1F). These results showed that the phenotype of the mutant was due to both cell elongation and division.

### Identification of the *OsGatB* gene

**Figure 2:**
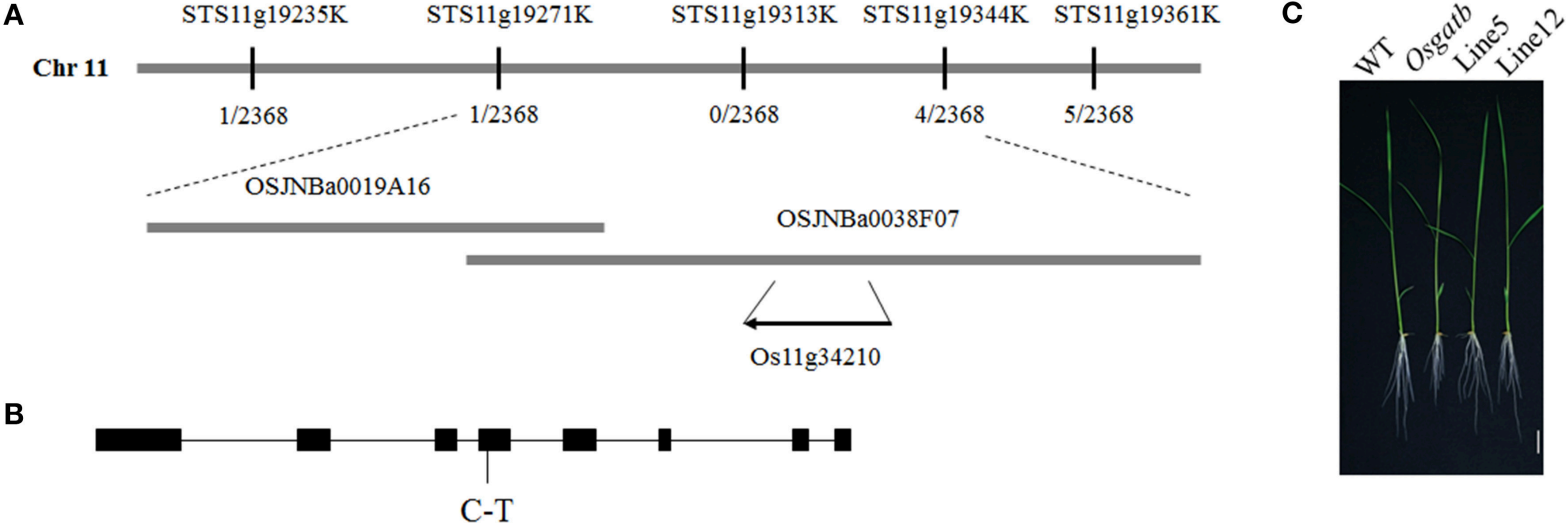
**Cloning of the *OsGatB* gene. (A)** Map-based cloning result of *OsGatB*. The *OsGatB* gene was identified as Os11g34210. **(B)** The gene structure of *OsGatB*. Black boxes indicate exons and lines represent introns. The point mutation is listed underneath. **(C)** Complementation analysis showed that the transgenic plants harboring the genomic DNA of *OsGatB* restored the short root phenotype of *Osgatb* mutant. Bar = 2 cm.

To determine the mutated locus in *Osgatb, Osgatb* was crossed with cv. Kasalath (*O. sativa* ssp*. indica*). According to map-based cloning and sequencing results, one point mutation was identified in the exon of GatB subunit family protein (LOC_Os11g34210; Figure 2A). The mutation changed C to T at 2412 bp, in the fourth exon of *OsGatB*, resulting in a change from Pro to Leu (Figure 2B).

To further confirm that the *OsGatB* mutation caused the *Osgatb* phenotype, one construct containing a 7.3-kb genomic DNA fragment including the coding region of the *OsGatB* gene and the promoter of *OsGatB* was transferred into the *Osgatb* mutant. A series of transformants displayed a WT phenotype (Figure 2C). Thus, phenotypic restoration confirmed that mutation of *OsGatB* was responsible for the short root phenotype.

### *OsGatB* is located in mitochondria

**Figure 3:**
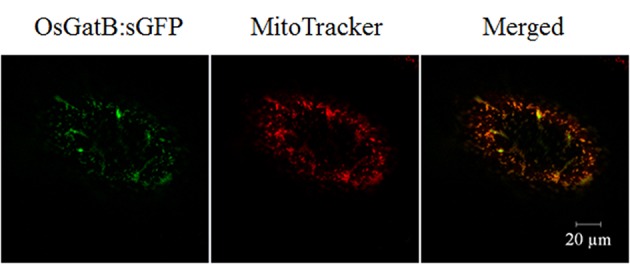
**Subcellular localization of OsGatB**. The OsGatB:sGFP fusion protein was located in mitochondria, co-located with a mitochondrial marker, MitoTracker, in onion epidermal cells.

The OsGatB protein predicted to have a mitochondrial targeting signal (http://psort.ims.u-tokyo.ac.jp/). The basic biochemical information of OsGatB was listed in Table S1.The orthologs of GatB, *Arabidopsis* GatB and yeast PET112 were identified as located in mitochondria (Pujol et al., 2008; Frechin et al., 2009). To further determine the localization of OsGatB, one constructor containing the coding region of OsGatB fused in-frame to synthetic green fluorescent protein (sGFP) was made and expressed in onion cells. MitoTracker was used as the mitochondrial marker (Carrie et al., 2009), and GFP in onion epidermal cells showed co-localization with MitoTracker (Figure 3). These results suggested that OsGatB was located in mitochondria.

### Expression pattern of *OsGatB*

**Figure 4:**
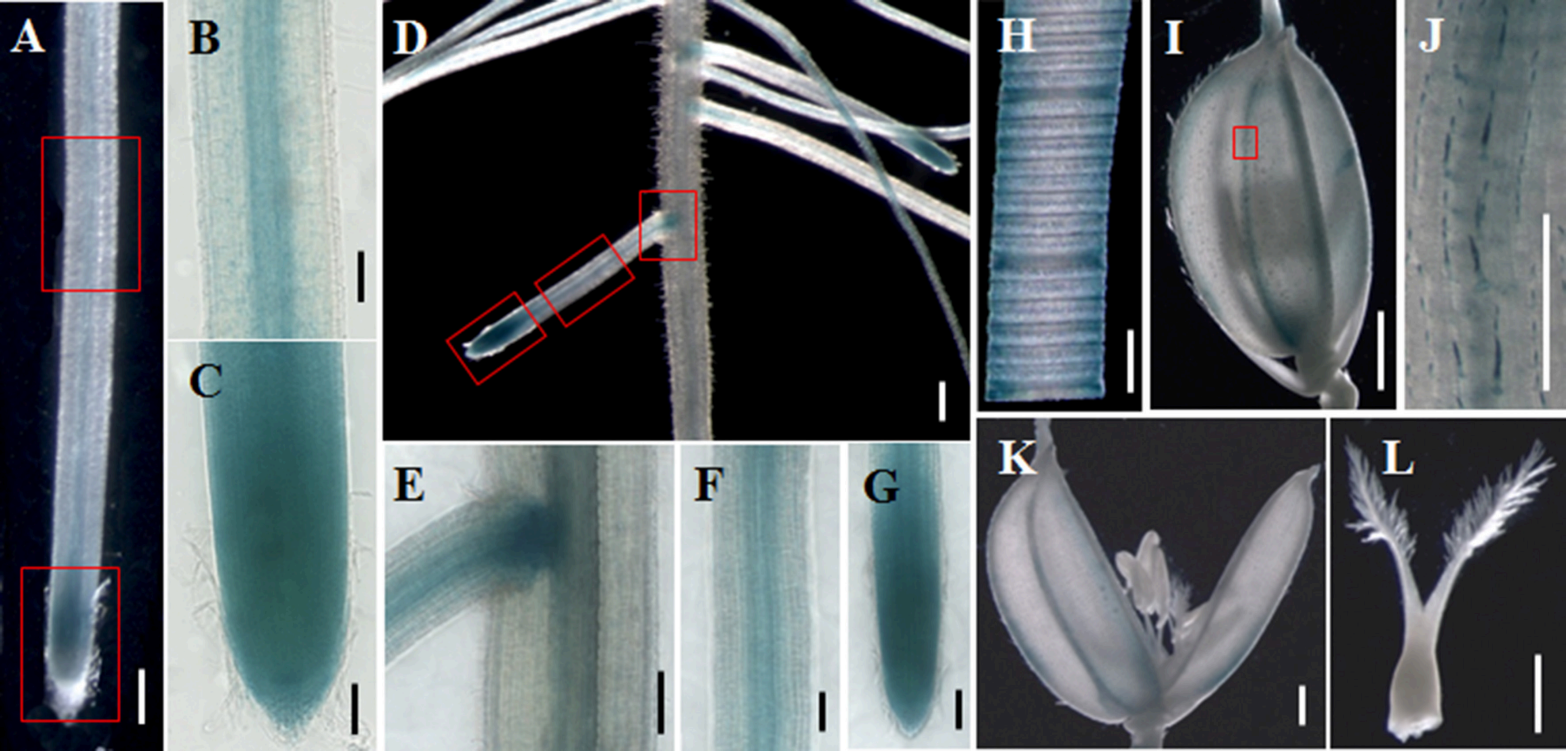
**Expression pattern of *OsGatB***. GUS staining of OsGatB–GUS in primary root (**A**), lateral root (**D**), leaf (**H**), spikelet **(I)** and flower **(K,L)**. **(B,C)**, **(E–G)**, and **(J)** are enlargements of the rectangles in **(A)**, **(D)**, and **(I)**, respectively. **(A,D)** Bar = 500 mm; **(B,C,E–I,K,L)** Bar = 200 mm; **(J)** Bar = 50 mm.

Quantitative real-time PCR analysis indicated that *OsGatB* was expressed in almost every plant organ, including roots, stem-base, stem, leaf, and panicle (Figure S2). To further examine the expression pattern of *OsGatB*, a fused gene of pOsGatB:OsGatB–GUS was developed. The fused gene rescued the *Osgatb* mutant, suggesting that the OsGatB–GUS protein acted as a functional protein, just as did the native OsGatB protein. GUS staining showed that *OsGatB* was expressed in almost all plant organs (Figures 4A–L). *OsGatB* was expressed in mature areas and root tips of primary and lateral roots, where mitochondria were present in large numbers.

### Mitochondrial ATP production, together with mitochondrial structure, are all affected in *OsGatB* root

**Figure 5:**
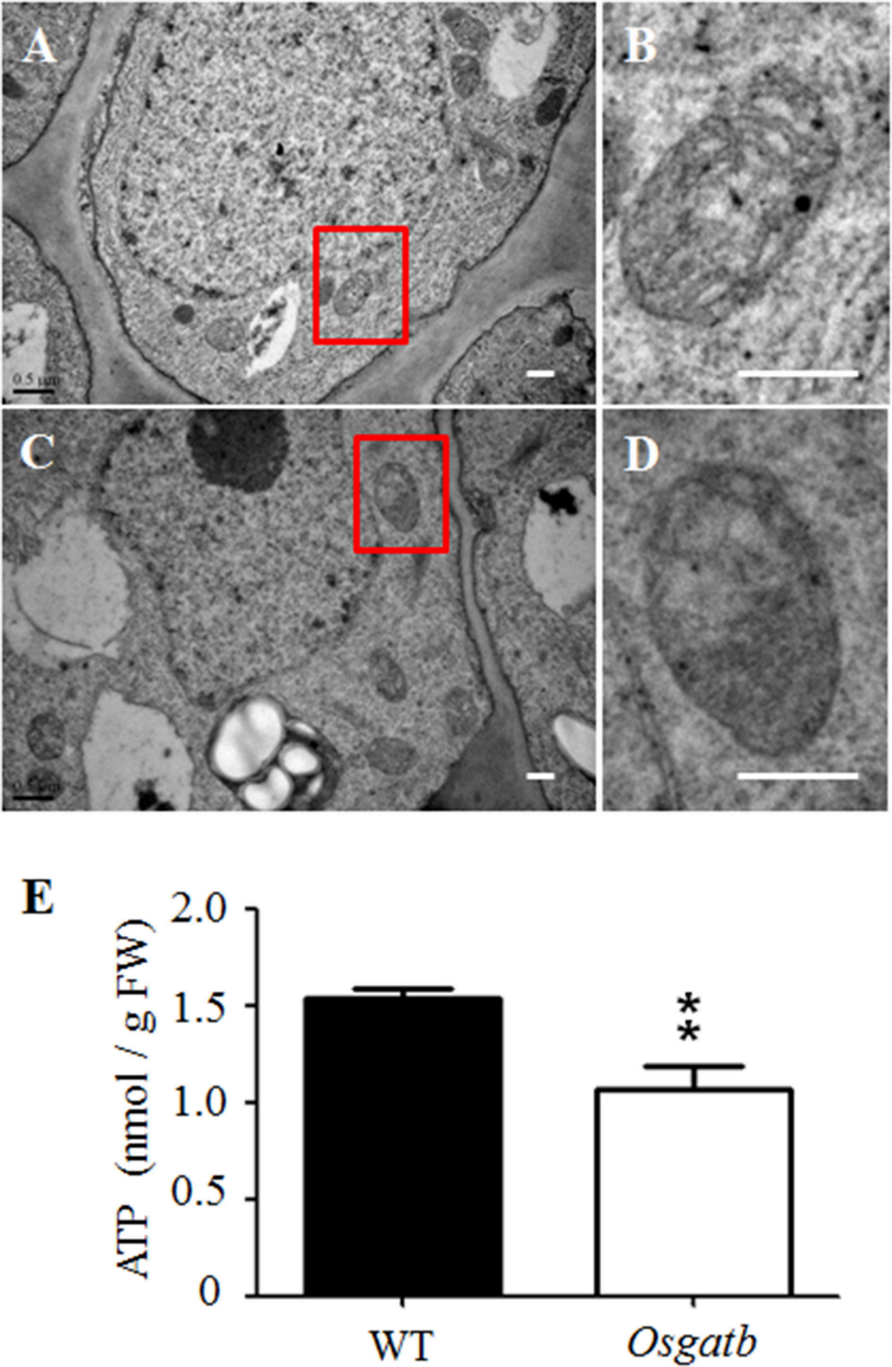
**Mitochondria structure and ATP content analysis**. Transmission electron micrographs of root tip of WT **(A,B)** and *Osgatb* plants **(C,D)**. **(B,D)** are enlargement of the rectangle in **(A)** and **(C)**, respectively. Bar = 0.5 μm. **(E)** ATP content in WT and *Osgatb* seedlings. The ATP level was lower in *Osgatb* than in WT plants. Error bars show the standard deviation from three independent experiments. Asterisks show significant difference between WT and mutant: ^**^P < 0.01. FW, fresh weight.

GatB was encoded by the nuclear genome and imported into mitochondria, to form the active AdT enzyme, which is involved in protein synthesis in mitochondria (Pujol et al., 2008). We therefore examined the distribution and number of mitochondria in the mutant root—there was no significant difference between WT and *Osgatb* mutant root tips. We then use transmission electron microscopy (TEM) to examine the mitochondrial ultrastructure in *Osgatb* and WT root tips. We analyzed the mitochondria in eight sections of WT and 11 sections of *Osgatb*, mitochondria in WT root tips were all of normal shape and contained an electron-dense matrix inside the outer membrane and well-developed inner membrane cristae (Figures 5A,B), but the majority (78%) of mitochondria in root tips from *Osgatb* plants were impaired (Figures 5C,D).

Since mitochondrial structure was altered in *Osgatb* root tip cells, we investigated whether mitochondria function was also altered. The energy from mitochondria is mainly used to synthesize ATP, so we measured the ATP levels in WT and *Osgatb* seedlings. The *Osgatb* mutants contained about 1.07 nmol ATP/g fresh weight (FW), which was 30.5% lower than in the WT (with about 1.54 nmol ATP/g FW; Figure 5E). Taken together, these results suggested that short root phenotype of *Osgatb* plants may be due to damage to mitochondrial structure and function.

## Discussion

In the present study, we isolated a short root mutant *Osgatb* and the mutant gene was identified as *OsGatB*, a gene encoding glutamyl-tRNA (Gln) amidotransferase B subunit family protein (Morgante et al., 2009). Gln-tRNA^Gln^ is essential for protein synthesis, and mutation of *GatB* in *Osgatb* plants may affect protein synthesis in root mitochondria. Many mitochondrial proteins play important roles in maintaining mitochondria structure and function, and mitochondria generate over 90% of ATP production, and play essential roles in determining cell division. We showed that *OsGatB* was located in mitochondria, and mitochondrial structure and function, especially ATP production, were all affected in *Osgatb* root tip cells (Figures 3, 5).

The hundreds of proteins needed to maintain the structure and function of mitochondria are encoded by the nuclear genome, but the proteins synthesized in the mitochondria are also involved in this process—these polypeptides are also essential for mitochondrial structure and function, although they are few in number. When mitochondrial translation is blocked, it causes lethality at very early stages. For example, when genes encoding mitochondrial aminoacyl-tRNA synthetases were mutated, the mutations were lethal during embryo development (Berg et al., 2005); mutations in mitochondrial ribosomal proteins can cause defects in gametophyte development (Portereiko et al., 2006). The fact that blocking mitochondrial translation is always lethal has greatly hampered research in this field. Fortunately, the mutation in this study—in *OsGatB*, a gene encoding the active AdT enzyme that is essential for protein synthesis in eukaryote mitochondria—is a point mutation producing a slight phenotype of short root, but was not lethal during the whole growth stage. This point mutation gives us a new pathway to study mitochondrial translation in plants.

The *GatB* subunits have been identified in some eukaryotic genomes. The *GatB* ortholog in yeast, *PET112*, is essential for mitochondrial functions (Mulero et al., 1994; Frechin et al., 2009). The AtGatB protein is located in mitochondria and is involved in Gln-tRNA^Gln^ formation in mitochondria (Pujol et al., 2008). GatB proteins are highly conserved in *Arabidopsis* and rice (Figure S3). In the rice genome, glutamyl-tRNA (Gln) amidotransferase B subunit family protein is encoded by only one gene, *OsGatB*. Consistent with this, we found that OsGatB protein localized in the mitochondria (Figure 3). Although, *OsGatB* was expressed in almost all plant organs (Figure 4), it showed strong expression in the root tip, where the content of mitochondria is high in plants. We also showed that mitochondrial structure and function, especially ATP production, were all affected in the primary root of *Osgatb* mutants (Figure 5). The expression pattern of *OsGatB* might explain the short root phenotype of the *Osgatb* mutant. In rapidly dividing and elongating tissues, like root tips, additional energy input from mitochondria is required. New energy is needed to promote the initiation of cell division and elongation, which need high ATP production (Elorza et al., 2004). Alternatively, decreased enzyme activity of OsGatB in the mitochondria caused lower ATP production in root tips, resulted in reduced cell division rate and finally impaired primary root growth.

Impaired mitochondria in the root tips of the *Osgatb* mutant suggests that mutation in *OsGatB* affects the structure and function of mitochondria, lead to energy deficiency and finally interrupt cell division in the root tips. It is important for future work to determine the molecular pathways through which *OsGatB* regulates mitochondria structure and function, and finally cell division and elongation in plants.

## Author contributions

CQ, LC, and PW conceived and designed the experiments. CQ, LC, HZ, MH, JS, and YZ performed the experiments. CQ, LC, and MH analyzed data. CQ, LC, and HZ wrote the manuscript. All authors read and approved the manuscript.

### Funding

This work was supported by the National Natural Science Foundation of China (Grant number 31500251), the Zhejiang Provincial Natural Science Foundation (Grant number LQ13C060003), and the China Scholarship Council (Grant number 201508330107).

#### Conflict of interest statement

The authors declare that the research was conducted in the absence of any commercial or financial relationships that could be construed as a potential conflict of interest.

## Abbreviations

AdT: tRNA-dependent amidotransferase
EMS: Ethyl methane sulfonate
FW: Fresh weight
GatB: Glutamyl-tRNA (Gln) amidotransferase B
GUS: β-Glucuronidase
sGFP: Synthetic green fluorescent protein
TEM: Transmission electron microscopy
WT: Wild type.
