# Supplementary material for: OsGatB, the Subunit of tRNA-Dependent Amidotransferase, Is Required for Primary Root Development in Rice
Source: Front Plant Sci. 2016 May 2;7:599. doi: 10.3389/fpls.2016.00599 (PMC4852291; doi:10.3389/fpls.2016.00599)
Supplement: Supplementary file 1 [file Table1.DOCX]

**Table S1.** **The biochemical data for OsGatB.**

|  |  |  |  | polypeptide | | |
| --- | --- | --- | --- | --- | --- | --- |
| Gene | Locus ID | ORF length(bp) | No. of introns | Length (aa) | Molecular mass (kDa) | pI (isoelectric point) |
| *OsGatB* | LOC_Os11g34210 | 1635 | 7 | 544 | 60.1 | 6.14 |
