# Supplementary figures and images for: OsGatB, the Subunit of tRNA-Dependent Amidotransferase, Is Required for Primary Root Development in Rice
Source: Front Plant Sci. 2016 May 2;7:599. doi: 10.3389/fpls.2016.00599 (PMC4852291; doi:10.3389/fpls.2016.00599)

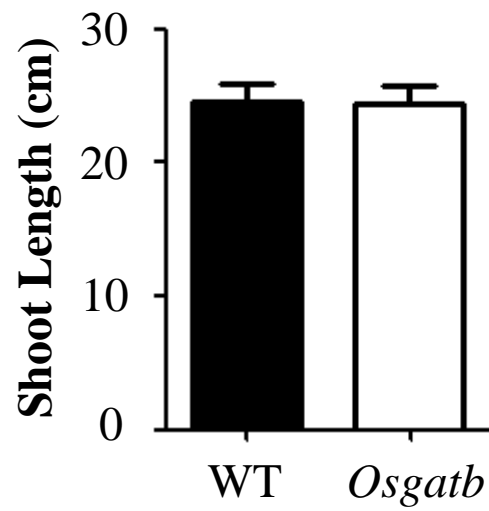

**Figure S1.** Shoot length of WT and *Osgatb* mutant.

Supplement: Supplementary file 2 [file Image1.PDF]

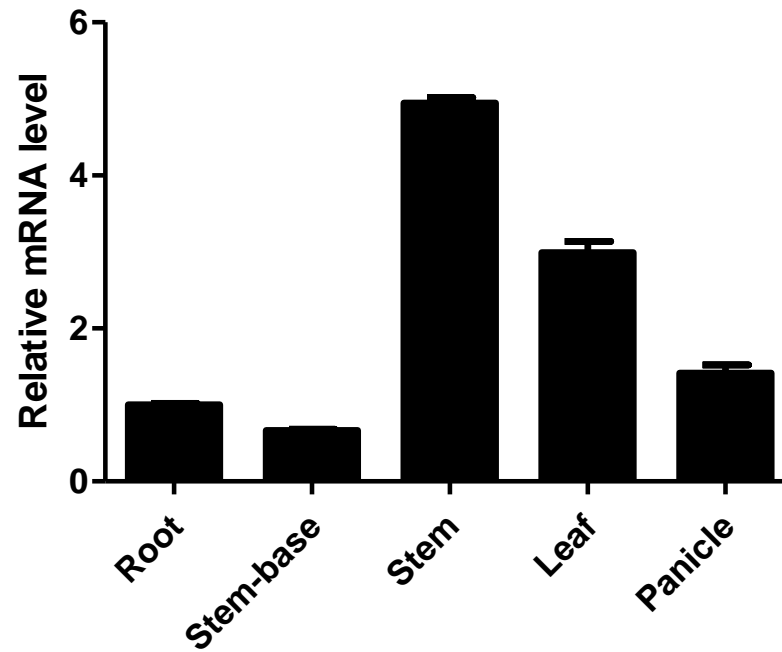

**Figure. S2.** The expression of *OsGatB* in the different organs.

Supplement: Supplementary file 3 [file Image2.PDF]
