# Supplementary material for: OsGatB, the Subunit of tRNA-Dependent Amidotransferase, Is Required for Primary Root Development in Rice
Source: Front Plant Sci. 2016 May 2;7:599. doi: 10.3389/fpls.2016.00599 (PMC4852291; doi:10.3389/fpls.2016.00599)
Supplement: Supplementary file 4 [file Image3.PDF]

```
AtGatB  MSTTLLRTIQLNQFSLLGTSLLRRRRSNNFSVRSCGSQTTTTHEAKQSSPTRVAPKNHKS
OsGatB  MALTLLRGMRTPVVARRNAGLFFTTLQSPLLSR....FTMRAESARAAAPKSIQLATKEA

AtGatB  NQLDEILRDYEAVIGIETHVQLSTLTKAFCSCSNNYGSYPNTSICPVCMGLPGALPVLNS
OsGatB  A..EQKAQGFEAVIGIETHVQLSTVTKAFCSCPYSYGSQPNSTVCPTCMGHPGTLPVLNA

AtGatB  KVVEFGVRLGLALNCDLSLKSKFDRKQYFYPDLPKGYQISQFDIPIASGGYVDVDIPLEF
OsGatB  KVVECAVRLGLALNCEIAMTSKFDRKQYFYPDLPKGYQISQFDIPIAKEGYLDLDLPVEF

AtGatB  GGGHRRFGITRVHMEEDAGKLLHSDTGDYSQVDLNRAGVPLLEIVSEPDMRSGIEAAEYA
OsGatB  GGGHRRFGVTRVHMEEDAGKLLHSESGSYSQVDLNRAGVPLLEIVSEPDMRTGIEAAEYG

AtGatB  CEMQRIARYLGVSNGNMQEGSLRCDVNISIRPIGQAEFGTKVEIKNLNAFSAISRAIDFE
OsGatB  AELQRLVRYLGVSNGNMQEGSLRCDVNVSVRPIGQSNFGTKVEIKNMNSFSAISRAIDYE

AtGatB  ISRQALLYNQGKADQIVTETRLWEEGAQKTVTMRKKEGLADYRYFPEPDLPEVILTQEYV
OsGatB  ISRQILLHKEGQADQIVQETRLWDESSQKTFTMRKKEGLADYRYFPEPDLPEVVLTSEYI

AtGatB  DSIRASLPELPEAKRRRYEAMGLGMQDVLFLANDVSVAEYFDAVIGKGAEVKLAANWIMS
OsGatB  DEIQNSMPELPEAKRRRFENMGLSMQDVLFLANDDNVARFFDSTLEHGADAKLAANWIMG

AtGatB  DIAAYLKNEKLSINDIKLTPQELAELIAAIKDGTISGKIGKEILFELLAKGGTVKGMIKA
OsGatB  DIAAYLKNEKLSIDEIKLTPLELSELIASIRNGTISGKIGKEILIELIAKGGTVKSVIEE

AtGatB  KDLVQITDPAEIEKMVIQVVSENPKQLEQYRSGKTKLQGYFAGQVMKMSKGKANPGLLNK
OsGatB  KDLVQIADPAAIEAMVDQVLADNPKQLEQYRSGKTKLQGFFAGQVMKASKGKANPVLLNK

AtGatB  ILLEKLNAKD
OsGatB  ILGEKLKANS
```

**Figure. S3.** Alignment of the conserved domains of OsGatB and its plant homolog AtGatB by use of the ClustalX 1.81 program.
